# The correlation between abnormal Krüger strict morphology and the sperm DNA fragmentation index

**DOI:** 10.5935/1518-0557.20240068

**Published:** 2024

**Authors:** Tamyres Souza Garcia Alvim Ranzato, Mariana Duque de Mello, Paula Fontoura Coelho de Souza, Lincoln Bastos Farias Junior, Luiz Felipe Bittencourt de Araujo, Ivan Andrade de Araujo Penna

**Affiliations:** 1 Fluminense Federal University Niterói, RJ, Brazil; 2 Sperm Bank of Rio de Janeiro, Rio de Janeiro, RJ, Brazil

**Keywords:** morphology, DNA fragmentation, spermatozoon, infertility

## Abstract

**Objective:**

This study aims to investigate whether alterations in sperm DNA fragmentation rates are more frequent when Krüger strict morphology is ≥4% (normal).

**Methods:**

The retrospective study included 132 participants from March 2020 to November 2021. Participants were divided into two groups based on the inclusion criteria: normal and abnormal Krüger strict morphology, with a mean age of 40 years. Seminal analyses were conducted following the guidelines outlined in the 6th edition of the Manual for Examination and Processing of Human Semen (2021). The sperm chromatin dispersion test was used.

**Results:**

The results did not reveal a statistically significant difference between Krüger strict morphology and Sperm DNA Fragmentation Index correlation (*p*<0.05) between the normal and abnormal morphology groups (*p*<0.05) and in Seminal Parameters. Sperm concentration is lower when Krüger strict morphology is < 4% (abnormal) (*p*=0.007).

**Conclusions:**

In conclusion, abnormal Krüger strict morphology does not have a higher predisposition to increased sperm DNA fragmentation.

## INTRODUCTION

According to the 6th edition manual World Health Organization (WHO, 2021), conjugal infertility is defined as the inability of a couple to conceive after one year of unprotected sexual intercourse. Studies reveal that conjugal infertility affects approximately 48.5 million couples worldwide, with the male factor responsible for 20-30%, contributing up to 50% in overall infertility cases (Agarwal *et al.*, 2015). Fertility decline has been increasing over the years and has been linked to worsening semen quality. However, the male factor is often overlooked during the evaluation of couple infertility (Esteves *et al.*, 2021).

As a standard diagnostic tool for male infertility cases, the conventional semen analysis evaluates macroscopic and microscopic aspects of semen. Among the various parameters assessed by the semen analysis, morphology has been of great interest. Human semen samples may contain different types of spermatozoa with deformities related to the head, midpiece, and tail, and they may be associated with defects in spermatogenesis as well as pathologies in the epididymis. Abnormal spermatozoa have lower fertilization potential, and depending on the alteration, they may also have fragmented DNA (WHO, 2021).

According to the 6th edition manual (WHO, 2021), studies show that a stricter morphological assessment predicts higher fertilization rates. In 1986, Krüger and colleagues proposed a new method for morphological evaluation (WHO, 2021). Krüger’s strict morphology criterion establishes that the percentage of sperm considered normal must be equal to or greater than 4%. Additionally, it is also possible to determine the proportion of morphological abnormalities: head with an oval shape and regular surface, midpiece and tail without abnormalities, as defined by the World Health Organization (WHO, 2021).

The main mechanisms leading to sperm DNA fragmentation are related to defective maturation, abortive apoptosis (within the testicle), and free radicals (in the male reproductive tract). In addition to these mechanisms, clinical risk factors, unhealthy lifestyle habits, and environmental factors also contribute to DNA fragmentation.

Evidence regarding the importance of sperm DNA integrity in fertilization, embryonic development, implantation, and pregnancy is growing. Furthermore, authors emphasize the need for further studies to elucidate the role of fragmentation tests in clinical practice. Such tests have become more frequent in clinical scenarios, including patients undergoing varicocelectomy, experiencing recurrent miscarriages, unexplained infertility, failures in assisted reproduction technology, and infertile men exposed to lifestyle-related risk factors (Cho *et al.*, 2017).

In this sense, the present study aimed to evaluate whether sperm DNA fragmentation is more frequent when Krüger strict morphology is equal to or greater than 4% (considered normal) in semen samples obtained from infertile men.

## MATERIAL AND METHODS

### Study Design

A retrospective study was conducted on 132 participants from the Sperm Bank of Rio de Janeiro (BSRJ) who underwent spermogram exams and sperm DNA fragmentation testing with up to 3 days of ejaculatory abstinence and presented normal seminal parameters according to seminal analysis (WHO, 2021). The study was conducted between March 2020 and November 2021. Participants were selected based on data collected from a questionnaire filled out by each participant before the sample collection. The questionnaire contained the participant’s medical history and the results of semen analysis and sperm DNA fragmentation. Exclusion criteria included participants with a history of testicular torsion, cryptorchidism, injury or cancer, mumps history, varicocele, diabetes, smoking, alcoholism, drug abuse, use of medications such as finasteride, presence of leukospermia, and recent fever history. Participants with professions exposed to environments and/or physical/chemical factors that could transiently impair spermatogenesis and consequently semen analysis (e.g., drivers, cooks, cyclists, farmers, and athletes) were also excluded (Jurewicz *et al.*, 2014; Jóźków & Rossato, 2017; Sunanda *et al.*, 2018). The participants were divided into two groups based on Krüger strict morphology: ≥ 4% (Normal) group, n=32, with a mean age of 41.00±4.66 years, and Krüger strict morphology < 4% (Abnormal) group, n=100, with a mean age of 40.73±5.67 years. The participants were evaluated in the study based on normal seminal concentration and progressive motility parameters, according to the criteria defined in the 6th edition of the World Health Organization manual (WHO, 2021).

This study complied with the following resolutions: Resolution nº 466 of December 12, 2012, and Resolution nº 411 of May 12, 2011, of the National Health Council (Conselho Nacional de Saúde, CNS, in Portuguese). The study was approved by the Research Ethics Committee of the Faculty of Medicine of the Fluminense Federal University (Universidade Federal Fluminense, UFF, in Portuguese), report number 5.376.567. The preparation of the study began in December 2021, started in May 2022 after the Committee approval, and concluded in November 2022.

### Seminal analysis

Before semen collection, patients were provided with instructions for the collection process. Following the instructions, a sterile, non-toxic container, appropriately labeled with patient data and the specific test to be performed, was provided for the sample collection. As the examination included a spermogram test with evaluation of sperm DNA fragmentation, the kit used (CANFrag - CANDORE BIOSCIENCE - India) for DNA fragmentation assessment recommends an ejaculatory abstinence period of up to 3 days, as per the kit’s standardization. The guidance and seminal analysis were conducted in accordance with the guidelines outlined in the 6th edition manual of 2021. The collection was performed in a designated room adjacent to the laboratory. Samples were obtained through masturbation. The collected sample was allowed to liquefy at room temperature (37°C), and both the seminal analysis (spermogram) and sperm DNA fragmentation assessment were performed within 30 to a maximum of 60 minutes after collection.

The seminal analysis and sperm fragmentation test were carried out by two observers from the laboratory of the Sperm Bank of Rio de Janeiro. Initially, macroscopic seminal parameters were analyzed, including volume, ejaculate appearance, liquefaction, viscosity, odor, and pH. Subsequently, the sample was prepared for microscopic analysis, ensuring thorough homogenization to guarantee the aliquots’ representativeness of the entire ejaculate.

Motility analysis, classification (rapid progressive, slow progressive, non-progressive, and immobile), and sperm concentration were performed using 10µl of the sample and Makler chamber under a light microscope with a x20 objective. The presence/absence of round cells, sperm agglutination, and vitality testing were also analyzed. Morphology evaluation involved preparing a smear with 10µl of the ejaculate sample on a slide. After drying and fixing, the slide was stained with Spermac Stain (FertiPro; Belgium). The morphology analysis was conducted using a light microscope with a x1000 magnification, and each observer evaluated 200 spermatozoa per participant whether they were ideal or abnormal, classifying their morphological alterations related to the spermatozoon’s shape (head, midpiece, and tail), according to the recommended classification.

### Sperm DNA Fragmentation Test

Sperm DNA fragmentation was made using the Sperm Chromatin Dispersion (SCD) test with the CANFrag Kit (CANDORE BIOSCIENCE - India). The method is based on the ability of intact sperm chromatin to form a halo of dispersion when exposed to acid and a lysis solution; the formed halos correspond to relaxed DNA loops linked to the residual nuclear structure, which are released after removal of nuclear proteins. DNA breaks, being susceptible to denaturation, prevent this dispersion (WHO, 2021). The CANFrag Kit principle is based on distinguishing between intact and fragmented sperm DNA.

The assay procedure consists of three main steps: 1) inclusion of a ejaculate sample aliquot in agarose gel to fix the spermatozoa on the slide; 2) addition of an acid denaturant, followed by a lysis solution to remove nuclear proteins; 3) washing with distilled water, dehydration in increasing ethanol baths, and finally, staining the slide for visualization under a microscope. Approximately 200 spermatozoa are evaluated to determine the percentage of spermatozoa with fragmented DNA. The interpretation of the result is based on the presence/absence of the dispersion halo observed on the slide. The percentage of DNA fragmentation is calculated by dividing the total number of fragmented spermatozoa plus degenerated spermatozoa by the total number of spermatozoa analyzed, multiplied by 100.

The DNA fragmentation index was calculated based on at least 200 spermatozoa per observer for each participant. Samples with high sperm concentration were diluted with sperm preparation medium, resulting in a sperm concentration of approximately 15 to 20 million/ml, following the instructions of the CANFrag kit. The reference value for the CANFrag Kit test is = 25%. Therefore, samples with a sperm DNA fragmentation index (SDF) < 25% are considered normal, while samples with SDF ≥ 25% indicate DNA fragmentation. According to the manufacturer, the CANFrag kit meets the requirements for accurate detection of DNA fragmentation in human spermatozoa, demonstrating stability and superior reproducibility above the minimum required.

### Statistical Analysis

The “Pearson’s Chi-squared test with Yates” was used to determine the association between Krüger strict morphology and Sperm DNA Fragmentation Index. The mean and standard deviation were calculated using the t-Student test, and the median (first and third quartile) was calculated using the Mann-Whitney test. We use these tests to evaluate the presence of significant differences between groups with different levels of sperm morphology in relation to DFI. We conducted normality tests and applied the appropriate tests based on the data distribution, as mentioned in the text. Numerical data were presented as mean ± standard deviation, frequencies as percentages, and minimum, mean, and maximum values were compared between two groups divided into Normal Krüger Strict Morphology (n=32) and Abnormal (n=100). Values were considered statistically significant at *p*<0.05. The R program, version 3.6.1, was used for the statistical analysis.

## RESULTS

In the present study, a total of 132 semen samples from 132 men from couples under infertility investigation were analyzed. Sperm DNA Fragmentation Indexes (DFI) (DFI ≥ 25% e DFI < 25%) were compared between two groups based on morphology: Krüger Strict Morphology <4% (abnormal) and Krüger Strict Morphology ≥4% (normal).

Among the participants with DFI ≥25% (altered), 22 had Krüger Strict Morphology <4% (abnormal), and 9 had Krüger Strict Morphology ≥4% (normal). Among the participants with DFI <25% (normal), 78 had Krüger Strict Morphology <4% (abnormal), and 23 had Krüger Strict Morphology ≥4% (normal).

Thus, the results did not reveal a statistically significant difference in the correlation between Krüger Strict Morphology and Sperm DNA Fragmentation Index, *p*=0.637 (Table 1). Out of the total participants analyzed, 32 had normal morphology, while the remaining 100 participants showed at least one morphological alteration. The test’s power resulted in 97%, indicating no association between Krüger Strict Morphology and Sperm DNA Fragmentation Index (Table 1).

**Table 1:** Fragmentation Index (DFI) comparison with two groups based on normal and abnormal Krüger Strict Morphology.

Krüger Strict Morphology	Total (n=132)	DFI ≥ 25% (n=31)	DFI < 25% (n=101)	^*^p value
Krüger < 4%	100	22 (22%)	78 (78%)	0.637
Krüger ≥ 4%	32	9 (28%)	23 (72%)	0.637

For Krüger Strict Morphology, Krüger ≥ 4% (Normal), Krüger <4% (Abnormal). For Sperm DNA Fragmentation Index (DFI), Sperm DNA Fragmentation Index ≥ 25% (Altered), Sperm DNA Fragmentation Index <25% (Normal).

* Statistically significant association, *p*<0.05. Pearson’s Chi-squared test with Yates.

The *p*-value mentioned in the text refers to the statistical significance of the correlation between Strict Krüger Morphology and the Sperm DNA Fragmentation Index (DFI). In this study, the *p*-value is 0.637. In other words, there is no statistically significant difference in the correlation between Strict Krüger Morphology and the Sperm DNA Fragmentation Index (DFI).

The demographic data of the groups are as follows: age, mean of 41,00 (standard deviation of 4.66) *vs*. 40.73 (standard deviation of 5.67); height, mean of 1.76 (standard deviation of 0.06) *vs*. 1.77 (standard deviation of 0.07); weight, mean of 90.22(standard deviation of 15.76) *vs*. 87.89(standard deviation of 14.09); BMI, 28.81 (standard deviation of 4.75) *vs*. 27.71 (standard deviation of 3.33); ejaculatory abstinence, 2.42 (standard deviation of 0.54) *vs*. 2.42 (standard deviation of 0.54) (Table 2). The *p*-value refers to the statistical test used to determine whether there are any significant differences in demographic data between the groups with normal and abnormal sperm morphology. These demographic data include age, height, weight, Body Mass Index (BMI), and ejaculatory abstinence. No statistically significant differences were found between the groups of normal and abnormal morphology, indicating that both groups are homogeneous (Table 2).

**Table 2:** Rate of development of transferred embryos.

Data	Krüger≥4%			Krüger<4%			*p*-value
	Minimum	Mean(σ)	Maximum	Minimum	Mean (σ)	Maximum	
Age (years)	34	41 (4.669)	55	23	40,73 (5679)	57	0.824
Height (m)	1.65	1.77 (0.067)	1.90	1.60	1.778 (0070)	2.00	0.551
Weight (kg)	62	90,22 (15.763)	157	68	87.89 (14.099)	140	0.183
BMI (kg/m^2^)	21.97	28.81 (4.755)	49,55	21.30	27.71 (3336)	38.58	0.325
Abstinence (days)	1	2.422 (0.540)	3	1	2.420 (0.549)	3	0.979

(σ) for standard deviation; for Krüger Strict Morphology, Krüger ≥ 4% (Normal), Krüger <4% (Abnormal); BMI, for Body Mass Index; Ejaculatory Abstinence, for the period of abstaining from ejaculation.

* Statistically significant association, *p*<0.05. The t-Student test was used for calculating the mean and standard deviation, and the Mann-Whitney test was used for calculating the median (first and third quartile).

When comparing the semen parameters (volume, pH, Concentration, and Progressive Motility) between two groups of participants with Krüger Strict Morphology <4% (abnormal) and Krüger Strict Morphology ≥4% (normal), the following results were obtained: semen volume, mean of 3.32 ml (standard deviation of 1.77) *vs*. 3.12 ml (standard deviation of 1.50); pH, mean of 8.15 (standard deviation of 0.14) *vs*. 8.11 (standard deviation of 0.19); Concentration, mean of 52.70x10^6^/mL (standard deviation of 33.62) *vs*. 28.82x10^6^/mL (standard deviation of 18.43); Progressive Motility, mean of 0.52% (standard deviation of 0.14) *vs*. 0.44% (standard deviation of 0.16) (Table 3).

**Table 3:** Seminal Parameters comparison with two groups based on normal and abnormal Krüger Strict Morphology.

Seminal Parameters	Krüger≥4%			Krüger<4%			*p* Value
	Minimum	Mean (σ)	Maximum	Minimum	Mean (σ)	Maximum	
Volume (mL)	0.8	3.322 (1.771)	8.80	1	3.127 (1.506)	9	0.685
pH	7.9	8.156 (0.145)	8.5	7.1	8.112 (0.192)	8.5	0.160
Concentration (10^6^/mL)	17.80	52.7 (33.629)	167.5	0.35	28.820 (18.436)	88	0.007
PM (%)	0.224	0.524 0.142)	0.8	0.00	0.449 (0.162)	0.756	0.525

(σ) for standard deviation; for Krüger Strict Morphology, Krüger ≥ 4% (Normal), Krüger <4% (Abnormal); PM for Progressive Motility.

* Statistically significant association, *p*<0.05. The t-Student test was used for calculating the mean and standard deviation, and the Mann-Whitney test was used for calculating the median (first and third quartile).

According to the text above, the *p*-value refers to the statistical significance of the comparisons between the semen parameters (volume, pH, concentration, and progressive motility) between two groups of participants with Krüger Strict Morphology <4% (abnormal) and Krüger Strict Morphology ≥4% (normal). Regarding the comparison of semen parameters: volume (ml), pH, and Progressive Motility (PM), no statistically significant differences were found between the groups of normal and abnormal morphology, indicating the presence of homogeneous groups. However, in terms of sperm Concentration (10^6^/mL), participants with Krüger Strict Morphology <4% (abnormal) have a lower sperm Concentration (10^6^/mL) compared to the group with Krüger Strict Morphology ≥4% (normal).

## DISCUSSION

In the present study, no statistically significant difference was observed in the correlation between Krüger’s Strict Morphology and the Sperm DNA Fragmentation Index (DFI), *p*=0.637 (statistically significant association, *p*<0.05). These findings support the results of other studies. Nguyen *et al.* (2022) found no differences in semen characteristics between the two groups of sperm DNA fragmentation, both in routine semen parameters and morphology abnormalities. Le *et al.* (2019) also reported no statistically significant correlation between semen parameters and the degree of sperm DNA fragmentation. Even in men with oligozoospermia, there was no correlation between DNA fragmentation and progressive motility, concentration, and morphology. These authors also did not find a correlation between DNA fragmentation and volume, concentration, and vitality when compared to morphology. Xie *et al.* (2018) after analyzing routine semen parameters in infertile men, did not find statistically significant differences between semen parameters and sperm DNA fragmentation. Each seminal parameter, including sperm morphology, showed no correlation with sperm DNA fragmentation index. Other authors, including Belloc *et al.* (2014), Mehdi *et al.* (2009), Sills *et al.* (2004) and Oliveira *et al.* (2010) also support this result.

This study is, for now, the second study conducted in Brazilians. Oliveira *et al.* (2010) also investigated the correlation between morphology and sperm DNA damage in Brazilians; however, their morphological analysis was performed using the examination of motile sperm organelles (MSOME), and DNA fragmentation was measured through TUNEL assays.

Among the DNA fragmentation evaluation methods mentioned at the beginning of the study - Transferase-mediated dUTP nick-end labeleling (TUNEL), Sperm Chromatin Structure Assay (SCSA), Sperm Chromatin Dispersion (SCD), and Comet assay (COMET) - we can say that in terms of assay sensitivity, the COMET method is the most sensitive, followed by TUNEL, then SCD, and SCSA with the lowest sensitivity (Ribas-Maynou *et al.*, 2013). The SCSA test has a standardized protocol, while the other tests vary considerably in methodology, making it difficult to compare their reproducibility; however, it should be noted that the COMET and SCD tests require less equipment robustness (Evenson, 2016).

Regarding the methodology used, our study differs from previously mentioned studies, which also differ from each other. The authors Nguyen *et al.* (2022) and Le *et al.* (2019) analyzed seminal parameters according to the WHO 2010 criteria (WHO, 2010), and the sperm DNA fragmentation index (DFI) was estimated using the sperm chromatin dispersion (SCD) assay, but the DFI threshold varied from 15% to 30%. The authors (Xie *et al.*, 2018) used the chromatin diffusion detection kit to assess DNA fragmentation through the Biosharp kit (Hefei, China). On the other hand, Belloc *et al.* (2014) and Mehdi *et al.* (2009) assessed morphology according to David’s classification and detected DNA fragmentation using the TUNEL assay.

In our study, we also compared seminal parameters and the sperm DNA fragmentation index in both groups of normal and abnormal morphology; however, we did not find statistically significant differences in terms of semen volume, pH, progressive motility (PM), and DNA fragmentation index (DFI). These results also support the findings of Nguyen *et al.* (2022), Ferrigno *et al.* (2021), and Belloc *et al.* (2014). However, sperm concentration was higher in participants with Krüger’s Strict Morphology ≥ 4% (normal) compared to the group with Krüger’s Strict Morphology <4% (abnormal). This finding is in line with the results of Nguyen *et al.* (2022).

We also analyzed the characteristics of age, height, weight, and BMI of the participants between the two groups of normal and abnormal morphology. Considering that these characteristics can affect semen parameters, including morphology, and sometimes sperm DNA fragmentation, previous studies (Jensen *et al.*, 2004; Le *et al.*, 2019; Gao *et al.*, 2021; Ruiz-Valderrama *et al.*, 2022) have pointed out the importance of considering them. However, we did not find statistically significant differences in these characteristics between the two groups. Nevertheless, it is worth mentioning that these participants are part of a categorically homogeneous group, as previously observed, with similar mean age, height, weight, and BMI in both normal and abnormal morphology groups. These homogeneous demographic and laboratory characteristics were not well defined and analyzed in previous studies, and when evaluated, like age and BMI, they varied with increasing age (Le *et al.*, 2019). However, in Nguyen *et al.* (2022), the data showed a very homogeneous mean for age and the evaluated semen characteristics (volume, pH, and Progressive Motility).

One strength of our methodology is that, unlike most other studies on this subject, we excluded participants who produced semen samples under circumstances that could introduce artifacts or uncontrolled changes in semen parameters and DNA integrity (e.g., prolonged abstinence above 3 days), participants with a history of testicular torsion, cryptorchidism, injury, or cancer, history of mumps, varicocele, diabetes, smoking, alcoholism, drug abuse, use of medications like finasteride, presence of leukospermia, history of fever in recent days, and occupational exposure. Additionally, the analyses were performed on raw, fresh semen samples without any interventions that could interfere with spermatozoa morphology and DNA integrity, such as Swim-Up, Density Gradient, Washing, and even temperature increase through provoked liquefaction in a water bath or heated plates. The study was conducted at a single center, with two different observers. For the morphological and DNA fragmentation analysis, at least 200 spermatozoa from each participant were observed by each observer. Sperm morphology was analyzed using Krüger’s Strict Criteria, following (WHO, 2021) guidelines, which were defined based on investigations of sperm morphology capable of penetrating cervical mucus and binding to the *zona pellucida*. However, this well-defined participant inclusion (inclusion criteria) reduced the sample size in our study, which is a limitation.

In the present study, we did not find a significant relationship between sperm DNA fragmentation and abnormal morphology. The studies conducted on the relationship between sperm DNA fragmentation and abnormal morphology have shown heterogeneity and conflicting findings so far. While some studies suggested a relationship between abnormal morphology and sperm DNA fragmentation, including Sedo *et al.* (2016), Campos *et al.* (2021), Jakubik-Uljasz *et al.* (2020), Ferrigno *et al.* (2021), others did not find a significant relationship, as mentioned in the previous discussion. It is important to note that the cutoff values for the Sperm DNA Fragmentation Index (DFI) were not identical among the previous studies, as well as the assays used to assess sperm DNA fragmentation. For instance, Ferrigno *et al.* (2021), used the TUNEL assay and adopted a DFI threshold of >15%; Jakubik-Uljasz *et al.* (2020) used the sperm nuclear dispersion test and adopted a threshold of 0-15% for low SDF levels, 16-30% for moderate levels, and >30% for high levels; other authors, such as Khalili *et al.* (2006), used the acridine orange test to assess DNA integrity and considered a high value starting from 30% DFI; Belloc *et al.* (2014) used the TUNEL assay and considered a high DFI threshold of >30%; Le *et al.* (2019) also considered a threshold of >30% but used the sperm chromatin dispersion (SCD) test to evaluate DNA integrity. Therefore, the >25% cutoff defined as the altered Sperm DNA Fragmentation Index through the chromatin dispersion test adopted in this study should be considered in specific studies to reach a conclusion.

## CONCLUSION

In conclusion, the present study found no correlation between abnormal Krüger’s Strict Morphology and a higher predisposition to increased sperm DNA fragmentation. However, further studies are needed to explore the correlation of other semen alterations with the risk of increasing sperm DNA fragmentation.
